# Optical Imaging of Trigeminal Ganglion Excitation Evoked by Electrical Stimulation of the Trigeminal Nerve

**DOI:** 10.7759/cureus.75522

**Published:** 2024-12-11

**Authors:** Tomoaki Ujita, Toru Yamamoto, Yurie Sato-Yamada, Naotaka Kishimoto, Takeyasu Maeda, Kenji Seo

**Affiliations:** 1 Division of Dental Anesthesiology, Faculty of Dentistry Graduate School of Medicine and Dental Sciences, Niigata University, Niigata, JPN; 2 Center for Advanced Oral Science, Niigata University Graduate School of Medical and Dental Sciences, Niigata University, Niigata, JPN

**Keywords:** di-4anepps, membrane voltage sensitive dye, optical imaging, peripheral nerve stimulation, trigeminal ganglion

## Abstract

Background

There are many reports of anatomical and physiological studies on trigeminal ganglion neurons, but few studies have analyzed temporal changes in the excitation of the trigeminal ganglion. This study aimed to establish an experimental system for spatial and temporal imaging analysis of the excitatory dynamics of trigeminal ganglion cells evoked by stimulation of a peripheral branch of the trigeminal nerve.

Methods

After excision of the trigeminal ganglion with the inferior alveolar nerve (IAN) from Sprague Dawley rats (seven to nine weeks old), 400-µm-thick slices of the trigeminal ganglion with the IAN were prepared. Real-time optical imaging was performed using cell membrane voltage-sensitive dye, Di4-ANEPPS, and changes in fluorescence intensity ratio (ΔF/F) were analyzed.

Results

Electrical stimulation of the IAN evoked the excitation of the trigeminal ganglion at the lateral surface area first, followed by expansion to the inner area. The calculated conduction velocity was 0.72 ± 0.49 m/s. This response was diminished by tetrodotoxin perfusion, but it was not observed in the C fiber-deficient rats.

Conclusions

A real-time optical imaging system can visualize the excitation of the trigeminal ganglion evoked by C-fiber stimulation.

## Introduction

The trigeminal ganglion consists of neurons and nonneuronal cells, and trigeminal neurons convey several types of sensory information from the oral and maxillofacial regions [1]. Many electrophysiological studies have shown the response dynamics of excitation of dissociated trigeminal ganglion cells [2], and morphological studies have confirmed the relationship between neurons and nonneuronal cells, such as satellite glia [3,4]. These studies also demonstrated that the excitation of trigeminal ganglion neurons is affected by neurotransmitters and neuromodulators [3,4]. However, it remains unclear how the activation of the axons affects the spatial and temporal excitation responses of neurons within the trigeminal ganglion.

Optical membrane potential imaging is a method for visualizing rapid changes in membrane potential by recording changes in fluorescence intensity, and it enables us to analyze the propagation of neuronal excitation within a slice preparation. It has been used to observe the propagation pattern of neuronal excitation in the central nervous system; however, to our knowledge, it has not yet been used to observe activity in the trigeminal ganglion [5].

To analyze how the sensory information from the three branches of the trigeminal nerve converges on the trigeminal root and whether signal modulation involves this structure, we applied optical imaging to visualize and characterize excitation propagation in trigeminal ganglion slices.

## Materials and methods

Animals

This is basic research conducted on trigeminal ganglion slices of eight rats at Niigata University, Niigata, Japan. All experimental procedures were approved by the Animal Research Committee of Niigata University (approval number SA00705) and conducted in compliance with the US National Institutes of Health (Bethesda, MD, USA) guidelines for the care and use of animals (NIH Publication No. 80-23, revised 1978). Male Sprague Dawley rats (neonatal or aged seven to nine weeks old; The Jackson Laboratory, Japan) were housed in pairs and maintained under a 12/12-h light/dark cycle at 25 °C with free access to food and water.

Slice preparation

**Figure 1:**
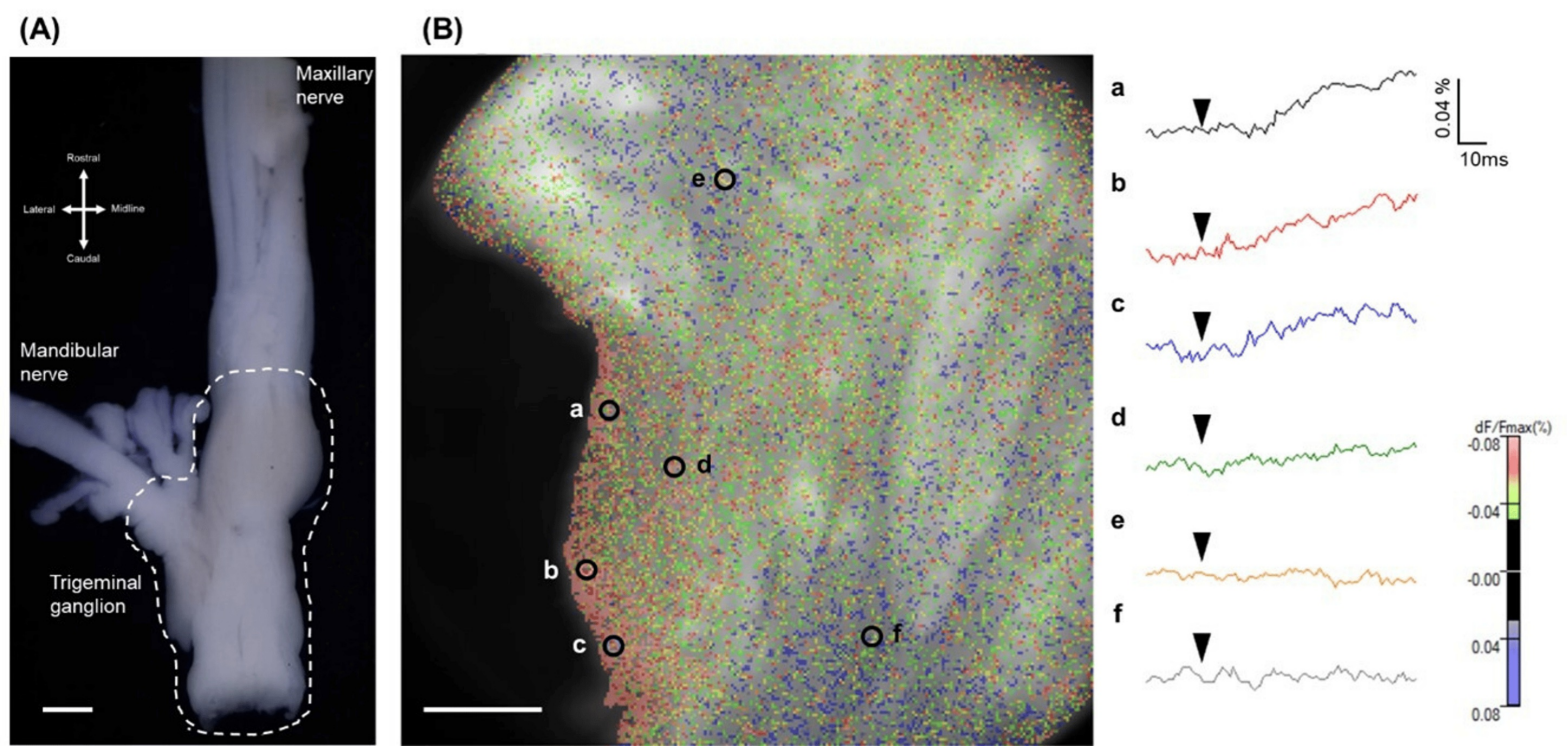
Slice preparation of trigeminal ganglion with the V3 and optical response of trigeminal ganglion slices produced by V3 trigeminal nerve stimulation (A) Dissected trigeminal ganglion (white dot line) with the V3. Obtained V3 is 20 mm in length. Scale bar: 1,000 µm. (B) Optical responses are indicated as red and green. Graph of optical reaction as the rate of change in fluorescence intensity (ΔF/F, %) through the recording time course at each point (a-f) within the trigeminal ganglion. Black arrows indicate the timing of electrical stimulation. Scale bar: 500 µm.

The rats were deeply anesthetized by sevoflurane inhalation, and medetomidine (0.375 mg/kg)-midazolam (2 mg/kg)-butorphanol (2.5 mg/kg) was injected intraperitoneally. The trigeminal ganglion and third branch trigeminal nerve (inferior alveolar nerve (IAN): V3) were quickly dissected (Figure 1A). The trigeminal ganglion was immersed in ice-cold artificial CSF (ACSF) equilibrated with a 95% O₂ and 5% CO₂ gas mixture. The composition of the ACSF was as follows (in mmol/L): 117 NaCl, 3.6 KCl, 1.2 NaH₂PO₄, 2.5 CaCl₂, 1.2 MgSO₄, 25 NaHCO₃, and 11 D-(+)-glucose. The dissected trigeminal ganglion was sectioned horizontally at a thickness of 400 µm using a microslicer (DTK-1000, Dosaka-EM, Kyoto, Japan). Sections were taken from a depth of 300-400 µm below the dorsal surface of the ganglion, which encompassed the V3. To stabilize the slices, they were perfused with ACSF at room temperature in a chamber (volume: 0.6 mL) for a minimum of 60 minutes. Staining of the slices was performed according to a previously reported protocol [6]. The slices were stained with 0.1 mM Di-4ANEPPS (Potentiometric Probes, Farmington, CT, USA) for 20 minutes at room temperature. The trigeminal ganglion and third branch trigeminal nerve (IAN: V3) were quickly dissected (Figure 1A). The trigeminal ganglion was immersed in ice-cold ACSF equilibrated with a 95% O₂ and 5% CO₂ gas mixture. The composition of the ACSF was as follows (in mmol/L): 117 NaCl, 3.6 KCl, 1.2 NaH₂PO₄, 2.5 CaCl₂, 1.2 MgSO₄, 25 NaHCO₃, and 11 D-(+)-glucose. The dissected trigeminal ganglion was sectioned horizontally at a thickness of 400 µm using a microslicer (DTK-1000, Dosaka-EM). Sections were taken from a depth of 300-400 µm below the dorsal surface of the ganglion, which encompassed the V3. To stabilize the slices, they were perfused with ACSF at room temperature in a chamber (volume: 0.6 mL) for a minimum of 60 minutes. Staining of the slices was performed according to a previously reported protocol [6]. The slices were stained with 0.1 mM Di-4ANEPPS (Potentiometric Probes) for 20 minutes at room temperature.

The stained slices were positioned in a measuring chamber (volume: 0.6 mL) on the stage of a fluorescence microscope and continuously perfused with ACSF (15-20 mL/min) saturated with 95% O₂ and 5% CO₂. The temperature of the ACSF was regulated at 24 °C during the course of the recordings. The tissue slice was subjected to excitation light at 530 nm, provided by a 30 W LED source (LEX3-G; BrainVision, Tokyo, Japan), and was imaged using a low-magnification objective lens (Plan APO; Olympus, Tokyo, Japan). The emission wavelengths transmitted through an optical filter (>580 nm) were detected using a tandem-type fluorescence microscope (THT Macroscope; BrainVision). Fluorescence images were acquired with a high-speed CMOS camera (MiCAM03-CMOS; BrainVision), and subsequent data analysis was conducted using MiCAM03 software (BV_Ana; BrainVision).

Each image was composed of 256 × 256 pixels, covering an area of 2.7 × 2.7 mm². A single-pulse electrical stimulation (2 mA, 500 µs) was applied to the distal end of the V3 using a small glass suction electrode fitted with a silver wire (Nilaco, Tokyo, Japan). Changes in fluorescence intensity induced by the stimulation were captured as 500 consecutive frames of fluorescence images, with a frame rate of 1,000 frames per second. For each optical imaging series, electrical stimulation was applied 32 times at 20-second intervals, and the resulting data were averaged to generate a set of 500 frames of fluorescence images. The fractional change in Di-4 ANEPPS fluorescence intensity for each pixel (ΔF/F), relative to the baseline fluorescence intensity prior to stimulation, was calculated and used as the optical signal. Since the same slice was imaged repeatedly, optical recordings were performed at 10-minute intervals.

To examine the anatomical consistency of where the fluorescence appears within the trigeminal ganglion when caused by specific peripheral nerve stimulation, we examined the spatial and temporal distributions of fluorescence within the trigeminal ganglion. To identify the type of nerve fibers that could display a fluorescence response, we analyzed the conduction velocity in the region that showed a fluorescence response based on the response latency and the length of the peripheral nerve (n = 6). Furthermore, to exclude neuron-derived optical responses in the wild-type group, tetrodotoxin (TTX; 206-11071, Fujifilm Wako Pure Chemical Co., Tokyo, Japan) was perfused (n = 3) and we examined the rate of change in the response area before and after perfusion. To compare the rate of change in the fluorescent response area in the capsaicin-treated group (n = 4) and the wild-type group (n = 3), the rate of change in response to a single stimulation was examined. The reaction area ratio was calculated by binarizing each image using ImageJ software (http://imagej.nih.gov/ij; provided in the public domain by the US National Institutes of Health), as described in a previous article [5].

Capsaicin treatment for neonatal rats

The procedures for the administration of capsaicin to neonatal rats and the dose of capsaicin administered in this study are described in the previous reports [7]. Capsaicin (030-11353; Fujifilm Wako, Osaka, Japan) was dissolved in an emulsion of 10% ethanol and 10% Tween 80 in isotonic saline for injection. Only male rats were used in this study.

Statistical analysis

The data are expressed as the means ± SDs or medians. Paired or unpaired t-tests were conducted to compare the responding areas in the optical analysis. Differences were considered significant at P < 0.05. All the statistical analyses were conducted using SigmaPlot (version 14.0; Hulinks, Tokyo, Japan).

## Results

Electrical stimulation of the distal end of the V3 afferent induced a characteristic spatial propagation of membrane depolarization in the trigeminal ganglion slice, and any were allowed for the period after the stimulus. Within the V3 region, the outer area corresponding to the V3 neuron-rich region exhibited high membrane excitation after stimulation (Figure 1B). The distribution of neurons outside the V3 base of the trigeminal ganglion was consistent with previous morphological reports. TTX abolished the excitation in the trigeminal ganglion (n = 3). The size of the depolarized area was significantly reduced in the presence of TTX than before TTX perfusion (paired t-test, P < 0.05). The ratio of the excitation area was 1.59 ± 0.15% and 0.98 ± 0.03% in the normal Krebs solution and TTX perfusion groups, respectively, at 60 ms after stimulation. The ratio of the excitation area was 1.743 ± 0.17% and 1.05 ± 0.21% in the normal Krebs solution and TTX perfusion groups, respectively, at 80 ms after stimulation.

The nerve conduction velocity was calculated from the latency of membrane excitation and the length of the V3. The mean (± SD) conduction velocity was 0.72 ± 0.49 m/s (n = 6), corresponding to the conduction velocity of C-fibers.

C-Fiber activation induced excitation in the preparation

**Figure 2:**
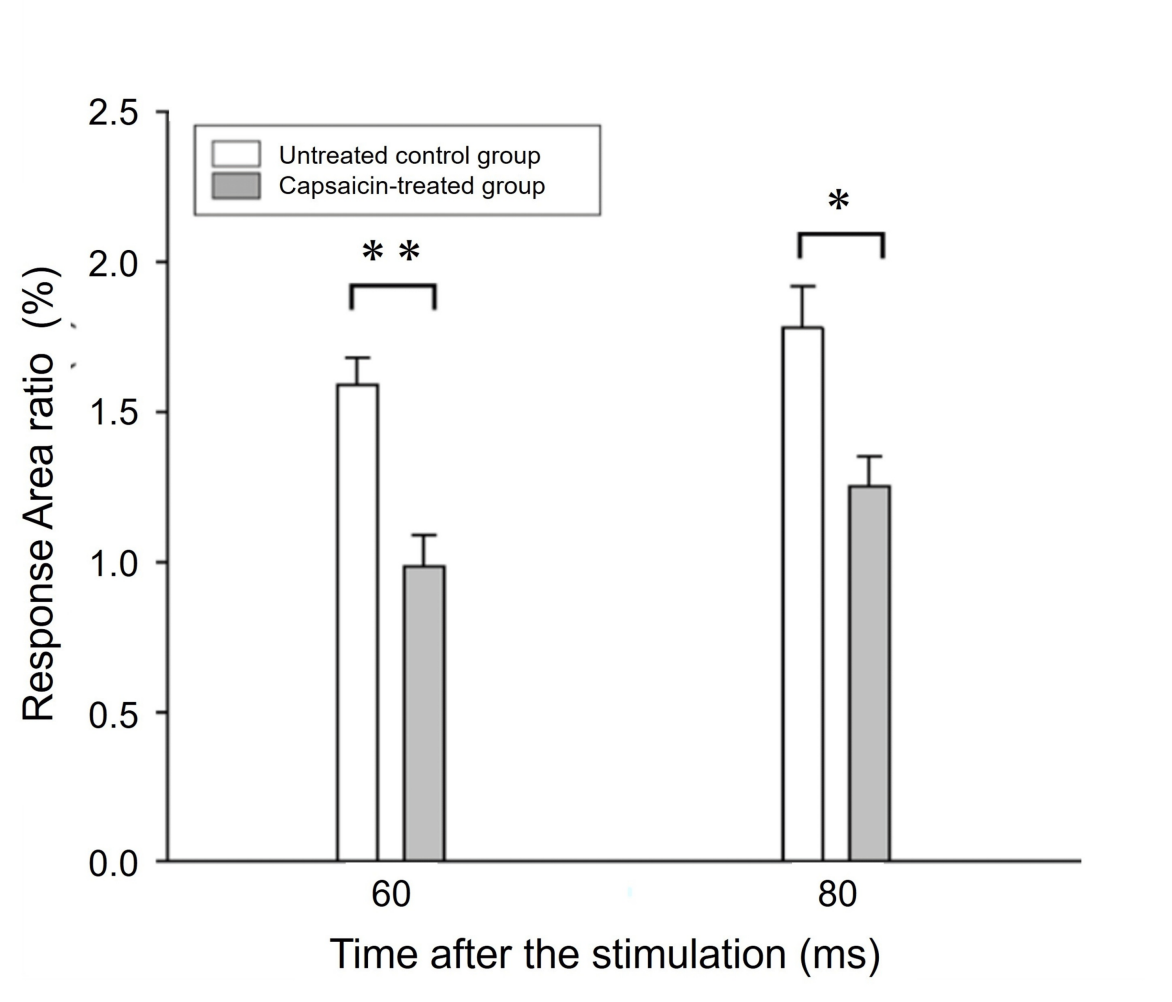
Comparison of optical response with capsaicin-treated C-fiber deficit rat model Comparison of optical response areas evoked by electric stimulation of the third branch of the trigeminal nerve in untreated control rats and capsaicin-treated rats. Note that capsaicin treatment reduced the ratio of optical responded area. * P < 0.05 ** P < 0.01, by t-test, untreated control rat n = 3, capsaicin-treated rat n = 4, respectively.

To confirm that C-fiber neurons produced the detected membrane excitation, we examined the optical imaging of membrane potential changes in rats whose C-fiber neurons were degenerated due to neonatal capsaicin treatment. In rats treated neonatally with capsaicin, a single stimulation evoked excitation propagation in the V3 region of the trigeminal ganglion; the ratio of the excitation area following 60 ms and 80 ms stimuli was significantly smaller compared to that of the untreated control rats at the same time points (P < 0.05, t-test) (Figure 2). These results indicate that optical imaging detected membrane potential changes in neuronal excitation in the V3 region that was meditated by C-fiber neurons.

## Discussion

This study succeeded in visualizing the propagation of neuronal excitation within the trigeminal ganglion slice evoked by electrical stimulation to the distal end of the V3 afferent. Furthermore, the voltage-gated sodium channel inhibitor TTX suppressed excitation within the ganglion slices, suggesting that the responses to stimulation were neuronal excitations evoked by direct stimulation of the trigeminal nerve afferent nerves. To our knowledge, this is the first visualization of peripheral nerve stimulation-mediated excitation and its propagation within the trigeminal ganglion.

We confirmed that the site responding to V3 stimulation within the trigeminal ganglion corresponded to the anatomical distribution of V3 neurons in the ganglion. Histological and retrograde tracer observations indicate that V3 neurons are located posterior to or at the root of the bifurcation of the V3 within the trigeminal ganglion [3,8]. In this study, primary afferent stimulation caused the neuronal cell excitation in the trigeminal ganglion, and the area of the excitation corresponded to the anatomical distribution of these studies. Furthermore, stimulation of V3 propagated from the outside of the ganglion to the inside of V3, but this response was not transmitted to the wider area of the ganglion. These findings also coincide with the fact that there is no synaptic transmission to another neuron within the ganglion. Therefore, the observed response was caused by cell excitation of the IAN.

Inhibition of voltage-gated sodium channels and the neonatal capsaicin-treated rat experiments indicated that C-fiber axons mediated neural activation. In this study, we utilized Di-4ANEPPS as a membrane potential-sensitive dye because it responds to changes with greater sensitivity than Ca2+ indicators [9]. Di4-ANEPPS can respond to changes in membrane potential with a time constant of less than 2 µs [10], allowing for rapid response observation. Moreover, the presence of the IAN in the longest afferent pathway among the trigeminal nerve branches means that electrical stimulation of the IAN has the longest latency of the optical response in the trigeminal ganglion.

While the latency of the A-fibers estimated from the mean conduction velocity was less than 1 ms, the minimum resolution bin in the measurement device was 1 ms, meaning that the reaction by the A-fibers could not be recorded. Therefore, the observed response of the trigeminal ganglion cells was mediated by a membrane potential change caused by C-fibers, not A-fibers.

A previous report [8] indicated that trigeminal neurons express various neuropeptides in the trigeminal ganglion and signal to neighboring neurons or satellite glial cells, which can signal to neurons with the same or other mediators. This process means that stimulated neuron excitation may also affect the glial cells surrounding the neurons. Therefore, in this study, the observation can include the mutual reactions of multiple cells, such as neuronal-glial cross-talk. Our observation that the optical response continued for a long time after the initiation of excitation is due to not only the excitation response of neurons that responded to stimuli but also the delayed excitation response of satellite glial cells via transmitters from those neurons. Another contributing factor is that, in this experiment, optical responses were assessed from a macroscopic perspective, encompassing three-dimensional cell populations, which may have resulted in the temporal overlap of individual cell responses. Previous studies examining the optical responses of single ganglion cells also reported relatively prolonged response times [10,11]. For instance, the assessment of neuronal activity using Di-4ANEPPS revealed that the activity decayed to the baseline at approximately 1,000 ms intervals [10]. Furthermore, studies that employed membrane potential dyes to observe the entire trigeminal ganglion similarly reported extended optical responses [12,13].

Ex vivo studies offer the advantage of enabling detailed analysis that cannot be observed in vivo studies by isolating the target from its surrounding tissue environment. In previous in vivo research on the trigeminal ganglion [12-14], observations were confined to the surface of the TG at the base of the skull, where neurons from the ophthalmic and maxillary branches are distributed [15]. However, neurons from V3 were difficult to observe, as they are located within the trigeminal ganglion inside the skull [16]. In this ex vivo study, by preparing slices of the trigeminal ganglion, we were able to analyze the excitatory dynamics of neurons associated with the V3 branch of the trigeminal ganglion. Furthermore, ex vivo studies have been employed in the development of therapeutic agents [16,17]. Consequently, this ex vivo approach holds significant potential for advancing the development of therapeutic drugs for trigeminal pain.

Recent advancements in research on trigeminal neuropathic pain have been facilitated by the use of animal models, such as those for trigeminal neuralgia and trigeminal nerve injury [18-20]. These models effectively replicate clinical symptoms commonly associated with neuropathies, including ectopic orofacial pain, mechanical allodynia, and mechanical hypersensitivity [18-20]. Given the significant impact of these symptoms on the quality of life in the maxillofacial region, it is crucial to elucidate the underlying pathological mechanisms of trigeminal neuropathic pain and to develop effective therapeutic approaches [21,22]. By applying this study to animal models, it is expected to offer valuable insights into the mechanisms by which primary neurons in the trigeminal ganglion modulate pain perception and sensory processing.

Limitations

This study has some limitations. We successfully detected the optical response of C fibers but were unable to detect the response of A fibers. This limitation is primarily attributed to the inadequate time resolution of the measurement equipment employed. Consequently, future investigations will require advancements in measurement technology to enable accurate detection of the A fiber response. Furthermore, to preserve the integrity of the peripheral nerves within the TG tissue slices, the thickness of the slices was constrained to approximately 400 μm. This restriction also limited the available cross-sectional area for optical observation. Finally, as this study focused on electrical stimulation, future research will aim to investigate the responses to alternative stimuli, such as pharmacological interventions or drug administration.

## Conclusions

This study established an ex vivo observation system for the excitation dynamics of trigeminal ganglion cells induced by stimulating a peripheral afferent branch of the trigeminal nerve.
